# Multistep cognitive behavioural therapy for eating disorders

**DOI:** 10.1186/2050-2974-2-18

**Published:** 2014-06-13

**Authors:** Susan M Byrne

**Affiliations:** 1 University of Western Australia, School of Psychology, Stirling Highway, Crawley 6009, Western Australia; 2 Center for Clinical Interventions, 223 James Street, Northbridge, Perth 6003, Western Australia

**Keywords:** Book review, Cognitive behavioral theraphy, Eating disorders

## Book details

Riccardo Dalle Grave, Jason Aronson, The Rowman and Littlefield Publishing Group Inc., Maryland, 2013, vii + 341 pp., ISBN: 978-0-7657-0927-1.

In the last 10 years, a new form of cognitive behavioural therapy for eating disorders, called Enhanced Cognitive Behavior Therapy (CBT-E) has been developed and tested by Christopher Fairburn and his colleagues at Oxford University. CBT-E derives from the ‘transdiagnostic’ theory of eating disorders [1], which developed out of the original cognitive behavioral theory of bulimia nervosa (CBT-BN; [2]). CBT-E represents a more potent, augmented form of CBT-BN which is able to be applied to all forms of eating disorder [1,3].

CBT-E has been evaluated empirically in outpatient populations in four randomized controlled trials [4-7] and in three community-based effectiveness trials [8-11]. CBT-E has also recently been evaluated in a randomized controlled trial of inpatient treatment for anorexia nervosa [9,10]. The results of these trials have provided evidence that CBT-E is well accepted by patients, even those who are severely underweight, and is highly effective for patients with all forms of eating disorders in a range of clinical settings.

This book describes the application of CBT-E to patients in different care settings requiring different levels of intensity of care (from out-patient to inpatient through to “intensive outpatient”). This approach, called Multistep Cognitive Behavioural Therapy for Eating Disorders, has been developed and described by Dr Riccardo Dalle Grave for use in his own eating disorder treatment program at the Villa Garda Hospital in Verona, Italy. Dr Dalle Grave has worked closely with Professor Fairburn and is well known for the contribution he has made to the theory and treatment of eating disorders over the past two decades.

This book is a very valuable companion to Fairburn’s comprehensive CBT-E treatment guideline, which was published in 2008 (“Cognitive Behavioural Therapy and Eating Disorders”). The book is divided into two main sections. In the first section, Dalle Grave’s model of applying CBT-E at three different intensity “steps” or “levels” according to the patients’ needs is described in detail and the rationale for his approach is clearly explained. The three intensity levels of CBT-E range from 1) outpatient CBT-E (CBT-E ‘as usual’) to 2) intensive outpatient CBT-E, which involves input from a multidisciplinary team including dietetics, a physician and a psychiatrist to 3) inpatient CBT-E. The guidelines for determining which level of care a patient requires, and when it is appropriate to step up or down from one level to another, is well set out in a format that is extremely user-friendly for clinicians and makes excellent sense.

The second section of the book consists of three detailed case studies of patients in each level of stepped care. The case studies include examples of conversations between therapist and patient illustrating various aspects of the treatment and beautifully illustrate the application of multistep CBT-E at each level and the specific treatment strategies that should be implemented as outlined in the first part of the book. They also make for fascinating reading!

I consider this book to be an excellent resource for clinicians, from any discipline, working with individuals with eating disorders. The chapters are all highly practical, as are the appendices which contain patient handouts and other resources (including useful internet sites). Overall, the book is well written, easy to follow and enormously practical. The style is always clear and concise.

## Competing interests

The author declares that she has no competing interests.
